# Icotinib-resistant HCC827 cells produce exosomes with mRNA *MET* oncogenes and mediate the migration and invasion of NSCLC

**DOI:** 10.1186/s12931-019-1202-z

**Published:** 2019-10-12

**Authors:** Yiming Yu, Maidinaimu Abudula, Chaofen Li, Zhongbo Chen, Yun Zhang, Yichen Chen

**Affiliations:** 1 The Affiliated Hospital of Medical School of Ningbo University, Ningbo, China; 2 Ningbo University, Ningbo, China; 3 Ningbo Ninth Hospital, Ningbo, China; 4 Ningbo Institution of Medical Science, Ningbo, China

**Keywords:** Icotinib resistance, Exosomal *MET*, NSCLC, Migration and invasion

## Abstract

**Background:**

Icotinib has been widely used in patients with non-small cell lung cancer (NSCLC), and have significantly enhanced the overall survival rate of NSCLC patients. However, acquired drug resistance limits its clinical efficacy. Tumor cell-derived exosomes have been reported to participate in various biological processes, including tumor invasion, metastasis and drug resistance.

**Materials and methods:**

In the present study, drug resistance was measured by MTT assay. Exosomes were extracted from the cell supernatant using ultracentrifugation and identified by exosomal marker. HCC827 cells were treated with exosomes derived from icotinib-resistant (IR) HCC827 to observe the invasion and migration of parent cells. The expression of exo-mRNA was analyzed by reverse transcription-quantitative polymerase chain reaction (RT-PCR). In addition, 10 exo-mRNAs detecting from the plasma and bronchoalveolar lavage fluid (BALF) of NSCLC patients with icotinib treatment were used to establish a new drug resistant-warning formula.

**Results:**

The oncogene *MET* into exosomes was identified from icotinib-resistant lung cancer cells, and this was also presented in exosomes in NSCLC patients diagnosed with cancer metastasis after icotinib treatment. The knockdown of *MET* in exosomes significantly decreased the ability of invasion and migration in HCC827 cells.

**Conclusion:**

It was suggested that *MET* might be specifically package and transferred by exosomes to modify the invasion and migration ability of the surrounding icotinib-sensitive cells.

## Background

Lung cancer is the leading cause of cancer death worldwide, and non-small cell lung cancer (NSCLC) is the most common histological type of lung cancer [1]. According to the epidemiological investigation by 2016, the number of patients with lung cancer in the United States has reached 222,500 [2]. Platinum-based combination chemotherapy has a survival rate of less than one year for the majority of patients with advanced NSCLC [3]. This disappointing result has prompted the search for new drugs and treatments. Furthermore, it has been reported that approximately 14% of NSCLC patients harbor mutations in epidermal growth factor receptor (EGFR), which is a receptor tyrosine kinase (RTK) [4]. In the past few decades, epidermal growth factor receptor-tyrosine kinase inhibitors (EGFR-TKIs), such as gefitinib, erlotinib and icotinib, have been the most commonly used as a targeted therapy for patients with different types of EGFR-mutated NSCLC, which are believed to be the cornerstone of combined treatment with other drugs. Regardless of the remarkable progress in EGFR inhibitor therapy, the resistance to EGFR inhibitors, either intrinsic or acquired, has become a major clinical problem, and the median progression-free survival (PFS) for patients remains only at approximately 10–13 months [5].

Exosomes are small membrane vesicles with a diameter of 30–120 nm, and are released into the extracellular milieu through various cell types under physiological and pathological conditions, including antigen presentation and infectious agent transmission. Exosomes mainly serve as mediators of local and systematic communication by sharing genetic information or functional protein, thereby contributing to tumor growth, metastasis, angiogenesis and drug resistance [6–8]. Several studies have reported that plasmatic exosomal proteins like CD91,CD317 and EGFR in NSCLC patients can be promising diagnostic biomarkers [9–12]. Furthermore, it has been showed in a study that with the release of exosomes by NSCLC A459 cells during cisplatin stimulation, the sensitivity of A459 cells to cisplatin has decreased. This process may have been mediated by the exchange of exosomal contents via cell-to-cell communication [13]. However, the involvement of exosomes in the development of resistance to icotinib and tumor metastasis in lung cancer cells remains unclear.

In aim of the present study was to investigate the potential role of exosomes derived from icotinib-resistant HCC827 (HCC827IR) cells in tumor cell migration and invasion.

## Materials and methods

### Patients and specimens

A total of 10 NSCLC patients with *EGFR 19del* mutation, who were in the Affiliated Hospital of Ningbo Medical School of Ningbo University (Ningbo, China) during the period of August 2017 and December 2018, were included into the present study. All patients have been primarily diagnosed in the above-mentioned hospital. The clinical specimens, including serum and bronchoalveolar lavage fluid (BALF), were collected at the time of primary diagnosis and after the treatment with icotinib within a follow-up period of 3–6 months. The clinical characteristics of these patients are presented in Additional file 1: Table S1. All procedures were approved by the Ethics Committee of the Affiliated Hospital of Ningbo Medical School of Ningbo University (Ningbo, China), and each patient provided an informed consent before the specimens were collected.

### Cell lines and cell culture

The human NSCLC cell line HCC827, which was sensitive to icotinib and contained an EGFR exon 19 deletion (DelE746-A750), and the human normal pulmonary epithelial cell line BEAS-2B were purchased from Nanjing Cobioer Biological Science (Nanjing, China). The HCC827IR cell lines (HCC827IR1 and HCC827IR2) were generated by repeated exposure of HCC827 cells to gradually increased concentrations of icotinib (Dalian Meilun Biotechnology Co., Ltd., China) for over six months and HCC827IR-1 clones were selected for subsequent experiments and referred to as HCC827IR. The HCC827IR cells were cultured in RPMI-1640 medium (Gibco, USA) supplemented with 10% fetal bovine serum (Gibco, USA), penicillin (100 U/mL) and streptomycin (100 μg/mL). Pulmonary epithelial cell lines BEAS-2B were cultured in BEBM complete medium (Nanjing Cobioer Biological Science, China). All cell lines were maintained in a humidified incubator at 37 °C with 5% CO_2_.

### Exosome isolation and identification

The HCC827 and HCC827IR cell lines were cultured in media with 10% exosome-free FBS (by ultracentrifugation for 12 h). After 48 h, the cell culture media was collected, and the exosomes were isolated from the cell supernatant by differential centrifugation, as previously described [14]. Finally, the concentration of the exosomal protein was determined using a BCA protein assay kit (Thermo Scientific, USA). Then, CD9, CD63 and CD81 (Cell Signaling Technology, Beverly, MA, USA) expression was measured using western blot analysis. The aliquots were stored at − 80 °C. The extracted exosomes and pellets were sent to Hibio Technology Co., Ltd. (Hangzhou, China) for transmission electron microscope (TEM) observation and validation, and the size distribution analysis. Thus, these exosomes were prepared for protein/RNA extraction, cell treatment, etc.

### Exosomes fluorescence assay

This assay was performed to verify the internalization of the labeled HCC827IR-derived exosome through HCC827 cells. First, the HCC827IR-exosomes were re-suspended in 500 ul of PBS in a 1.5 ml microcentrifuge tube (Eppendorf, EP), and DiR iodide (Dalian Meilun Biotechnology Co. Ltd., China) was added to the tube with the HCC827IR exosome up to a final concentration of 5 μg/ml. Then, the mixture was incubated at 37 °C for 30 min without shaking. Afterwards, the EP tube was centrifuged at 1000 rpm for three minutes, and the supernatant was carefully filtered with a 0.22-μm filter. Subsequently, the HCC827IR-Exosome-DiR liquid was co-cultured with HCC827 cells for 24 h. Finally, these cells were observed under a fluorescence microscope.

### MTT assay

Cell activity was determined using the MTT assay. Cancer cells were seeded on 96-well plates at a density of 1 × 10^5^ in each well. After 24 h, these cells were treated with different concentrations (0, 2, 4, 8, 16 and 32 uM) of icotinib for 48 h. Then, a 15-μl MTT solution (0.5%) was added to the medium, and incubated for four hours at 37 °C. Afterwards, the medium was carefully removed, 150 μl of dimethyl sulfoxide (DMSO) was added to each well to dissolve the insoluble formazan product, and the absorbance of the colored solution was measured at 490 nm, calibration read at 630 nm using a microplate reader (MK3, Thermo, USA). Next, the background absorbance of the medium in the absence of cells was subtracted. All experiments were independently performed in quintuplicate, and the mean for the experiment was calculated.

### Western blot

The total proteins of cells and isolated exosomes were lysed with RIPA buffer supplemented with proteinase inhibitors (Beyotime Biotechnology, China), according to manufacturer’s protocols, and centrifuged at 14,000×g for 10 min at 4 °C. Then, the protein concentrations were determined using BCA protein assay kit. The protein(25μg of protein isolated from cells and 35μg of protein isolated from exosomes) were separated using 10–12% sodium dodecyl sulfate-polyacrylamide gel electrophoresis (SDS-PAGE), and transferred onto polyvinylidene difluoride (PVDF) membranes. The membranes were blocked with 5% skim milk within TBST for one hour at room temperature, and incubated with the primary antibodies at 4 °C overnight. The primary antibodies used for the western blot were as follows: anti-CD9 (1:1000; CST, USA), anti-CD63 (1:1000; CST, USA), anti-CD81 (1:1000; CST, USA), anti-GAPDH (1:3000; Bios, China), anti-EGFR (1:1000; CST, USA), anti-p-EGFR(1:1000; CST, USA), anti-AKT (1:1000; CST, USA), and anti-alpha-Actinin (1:1000; CST, USA). After incubation with the appropriate horseradish peroxidase-conjugated secondary antibodies, the immune-reactive protein bands were visualized with chemiluminescence reagents (CST, USA), followed by imaging on an electrophoresis gel imaging analysis system (D-Digital, USA).

### Cell migration assay and wound-healing assay

Cells were treated with exosomes (20 μg/ml) or culture medium without exosomes for the appointed time periods. For the cell invasion and migration assay, about 4 × 10^4^ cells were plated into the upper chamber that were pre-coated with or without Matrigel (Matrigel, BD, USA) The culture medium in the upper chamber was FBS-free RPMI 1640, while the medium in the lower chambers was the RPMI 1640 supplemented with 10% exosome-free FBS. After 24 h, all cells that had transferred to the lower chambers were fixed and stained with 0.5% crystal violet. Then, positive staining cells from six representative fields of chambers in each group were photographed and counted under a microscope (Nikon, Japan). For the wound-healing assay, equal numbers of cells pre-treated with or without exosomes were plated into six-well plates. Then, the cell monolayers were wounded with a pipette tip to draw a gap on the plates. HCC827 cells that migrated into the cleared section were observed under a microscope (Nikon, Japan) after 24 and 48 h.

### Cell cycle assay

Equal numbers of cancer cells were seeded and cultured in the bottom of 6-well plates overnight. Then, these cells were co-cultured with HCC827 or HCC827IR cells. After 48 h, cells at the bottom were collected and re-suspended in pre-cold PBS. Afterwards, the re-suspended cells were fixed with 70% cold alcohol for one hour, the suspension was centrifuged, and the cell particles were cultured in PI/RNase staining buffer for 15 min (BD Biosciences, CA, USA). Subsequently, the flow analysis of the stained cells was performed using a C6 flow cytometer (BD Biosciences).

### Immunofluorescence analysis

Cells were seeded in six-well plates and cultured for 24 h before being fixed by 4% paraformaldehyde, and incubated with specific primary antibodies vimentin (1:100; AF1975, Beyotime) and cytokeratin-7 (1:100; AF1822, Beyotime, China) at room temperature for one hour. Then, these were incubated in the dark with secondary antibodies against vimentin and cytokeratin-7. The staining was developed using a fluorescence detection system (Beyotime, China). The samples were counterstained with 4′,6-diamidino-2-phenylindole (DAPI, Invitrogen) to visualize the cell nuclei. After washing, representative images were examined under a fluorescence microscope (Leica, FL, USA).

### RNA extraction and quantitative real-time polymerase chain reaction (qRT-PCR)

Total RNA was extracted from cultured cells and purified exosomes using TRIzolTM reagent (Life Technologies, USA), according to the manufacturer’s instructions. The RNA was resuspended in RNase-free DEPC-water and stored at − 80 °C. An equal amount of RNA was used for reverse transcription. The cDNAs were synthesized by using a reverse transcription kit, according to manufacturer’s instructions (CWBio, Beijing, China). qRT-PCR for cellular/exosomal mRNA, including *CDK1* (Cyclin-dependent kinase 1)*, CDK2* (Cyclin-dependent kinase 2)*, CDK4* (Cyclin-dependent kinase 4)*, CDH1* (Cadherin 1)*, ICAM1* (Intercellular cell adhesion molecule-1)*, ITGB1* (Integrin beta-1)*, ITGB3* (Integrin beta-3)*, CDC42* (Cell division control gene-42)*, GAS6* (Growth arrest-specific 6) and *MET* (hepatocyte growth factor receptor)*,* as well as internal reference *GAPDH* (glyceraldehyde-3-phosphate dehydrogenase) were performed using RT-PCR Quantitation Kit (CWBio, Beijing, China) according the manufacturer’s instructions. Briefly, after an initial denaturation step at 95 °C for 10 min, the amplifications were carried out with 40 cycles at a melting temperature of 95 °C for 15 s, and an annealing temperature of 60 °C for 35 s. The relative expression levels of mRNAs were calculated with 2^–ΔCt^ method. PCR productions were tested by 2% agarose. The sequences of the specific primers are presented in Additional file 1: Table S1.

### IR exosome electroporation with *MET* siRNA

Next, Mixed 7.0 μg of IR exosomes with 0.33 μg of *MET* siRNA in a citrate buffer solution (prepared with DEPC water), with a final volume of 150 μl. The process of electroporation was performed according to the instructions provided by Scientz-2C (Scientz, Ningbo, China). Size exclusion chromatography (SEC) was performed to wipe off the redundant siRNA. Then, quantitative reverse transcription–polymerase chain reaction (RT–qPCR) were performed to detect the level of *MET* in IR exosomes.

### Statistical analysis

The statistical analysis was performed using the SPSS software (version 20.0; IBM Corp., Armonk, NY, USA) and GraphPad Prism version 6.0 (GraphPad). Comparisons between pairs were performed using Student’s *t*-test, while multiple comparisons between the groups were analyzed using one-way analysis of variance, followed by Student Newman-Keuls test. All experiments were performed in triplicate, and the results were presented as the mean ± standard deviation. *P* < 0.05 was considered statistically significant.

## Results

### Establishment of the icotinib-resistance HCC827 IR cell model

**Fig. 1:**
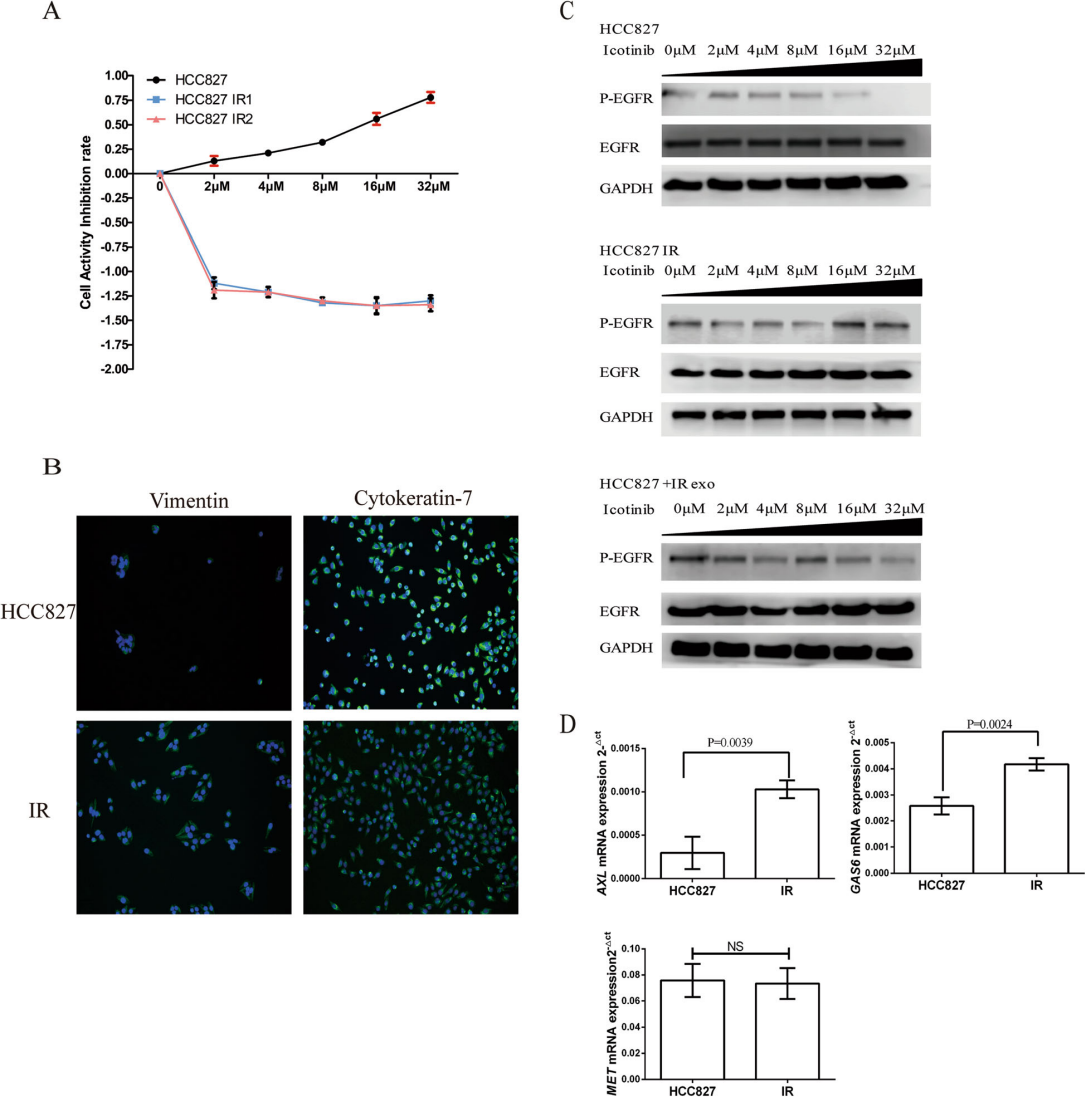
**a** The MTT assay of HCC827, HCC827IR1 and HCC827IR2 cells at the indicated concentration for 48 h. **b** The vimentin and cytokeratin-7 expression in HCC827 and HCC827IR cells were identified by immunofluorescence analysis. **c** The western blot analysis of EGFR and P-EGRF expression in HCC827, HCC827IR and HCC827 cells treated with IR exosomes. **d** The expression of *AXL*, *GAS6* and *MET* correlated to epithelial-mesenchymal transition (EMT) detected by qRT-PCR

Human NSCLC HCC827 cells with the deletion of exon19 of *EGFR* was used to establish the new model of acquired resistance to icotinib. These HCC827 cells were initially sensitive to icotinib treatment (the half inhibitory concentration [IC_50_] of 8.1 μM), and were cultured in the presence of icotinib, with an indicated concentration gradients. The HCC827IR clonal sublines were generated (HCC827IR1 and HCC827IR2) with icotinib IC_50_ > 80 *u*M through prolonged exposure time (for > 6 months) (Fig. 1a). In this study, the expression of vimentin and cytokeratin-7 was determined using immunofluorescence imaging, and it was found that fluorescence expression of vimentin was stronger in HCC827 IR cells, when compared with parental cells (HCC827) (Fig. 1b). For the subsequent experiments, HCC827IR cells were intermittently treated with 50 uM of icotinib to sustain its drug resistance feature. In order to determine whether these HCC827 cells sustained the activation and phosphorylation of EGFR after icotinib treatment, the phosphorylation status of EGFR was assessed. In cells treated with a certain concentration gradient of icotinib for 48 h, phosphorylation of EGFR was reduced with the increasing level of icotinib concentration (Fig. 1c). The western blot analysis confirmed that the activities of p-EGFR was inhibited by icotinib in a dose-dependent manner. Furthermore, in the context of acquired resistance to icotinib, the expression of phosphorylated EGFR in the HCC827IR cell line was detected by western blot. It was found that the expression of phosphorylated EGFR was not reduced with the increasing concentration level of icotinib, suggesting that phosphorylated EGFR was probably no longer the icotinib target in the HCC827IR model (Fig. 1c).

According to the microarray result from previous studies [15], three mRNAs (*AXL*, *GAS6* and *MET*) correlated to EMT were chosen for detection by qRT-PCR, and to whether these were differentially regulated in HCC827IR cells, when compared with parental HCC827 cells (*P* < 0.05). It was observed that the receptor tyrosine kinase *AXL* and its ligand *GAS6* were highly expressed in cells with acquired icotinib-resistance, while the expression of *MET* was the same both in parental cells HCC827 and HCC827IR cells (Fig. 1d).

### The co-culture with HCC827 IR improved the ability of migration in HCC827

**Fig. 2:**
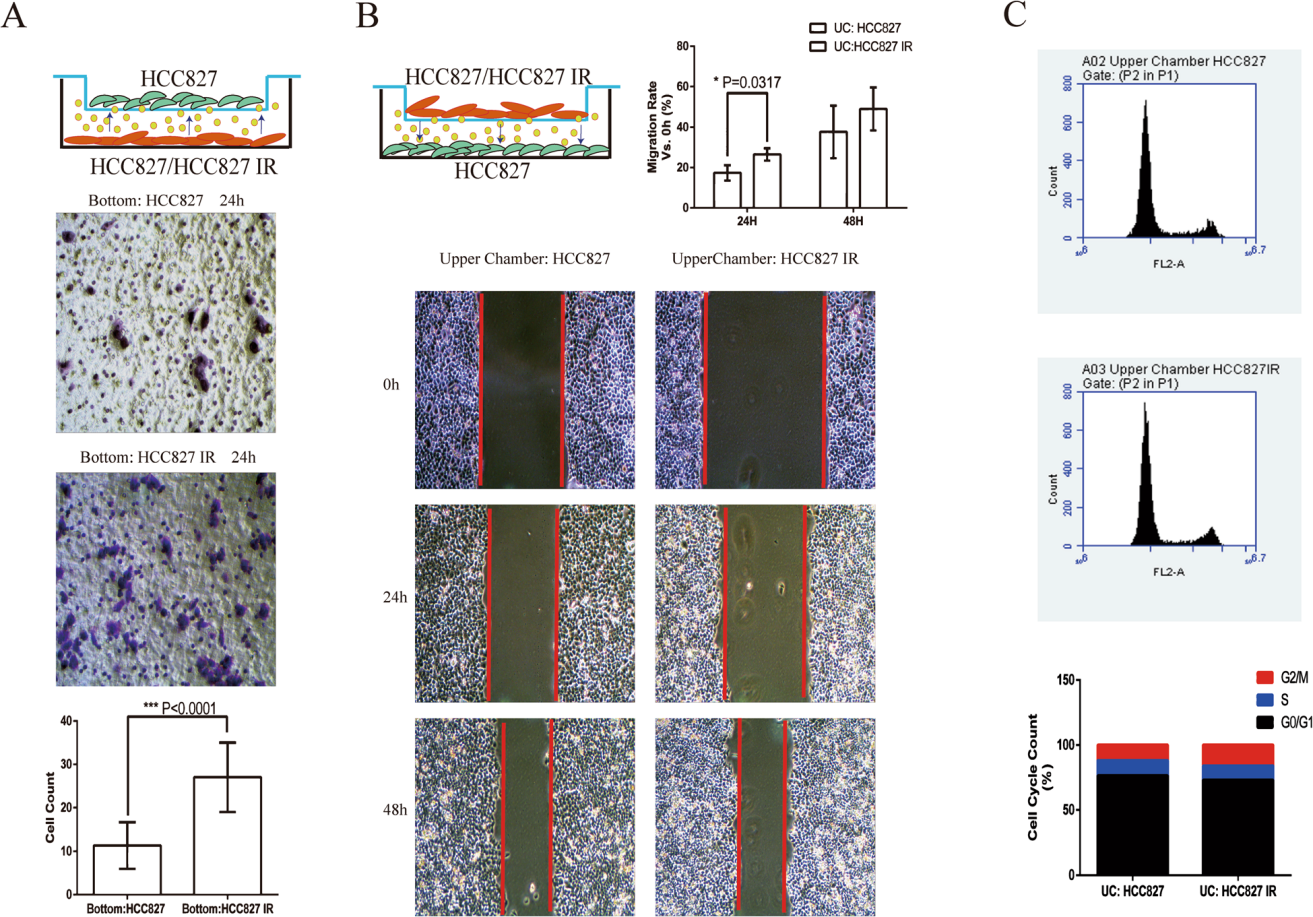
HCC827IR improved the migration ability of HCC827 cells. HCC827 cells were co-cultured with HCC827IR for 24 or 48 h. **a** The Transwell assay was performed to analyze the cell migration. The cell number of HCC827/HCC827IR was 29.25 ± 1.51 vs. those of HCC827/HCC827 was 10.61 ± 3.81)(*P* < 0.0001) (**b**) The cell migration was analyzed by wound-healing assay. The migration rate of HCC827IR/HCC827 in 24 h was 26.5% ± 1.765%, and the migration rate of HCC827/HCC827 in 24 h was 17.37% ± 2.206%, *p* = 0.0317. **c** The cell cycle was analyzed by flow cytometry

In order to determine whether the acquired icotinib-resistant cells have a potential effect on parental cells, HCC827 cells were co-cultured with HCC827IR cells, and HCC827 cells cultured in normal culture medium were used as a control group, and placed in a 6-well plate for 48 h, as previously described. The Transwell assay further confirmed that more HCC827 cells migrated in the HCC827IR affected group (the cell number of HCC827/HCC827IR was 29.25 ± 1.51 vs. those of HCC827/HCC827 was 10.61 ± 3.81)(*P* < 0.0001)(Fig. 2a). The wound-healing assay revealed that the HCC827 cells (bottom of the plate), which were co-cultured with HCC827IR, expressed a higher level of migration ability, when compared to the control group in 24 h (*P* = 0.0317) (Fig. 2b). Nevertheless, the cell cycle and cell apoptosis determined by flow cytometry revealed no significant difference between cells co-cultured with HCC827IR cells and the control group (Fig. 2c).

### HCC827IR cell-derived exosomes can be taken up by HCC827 cells

**Fig. 3:**
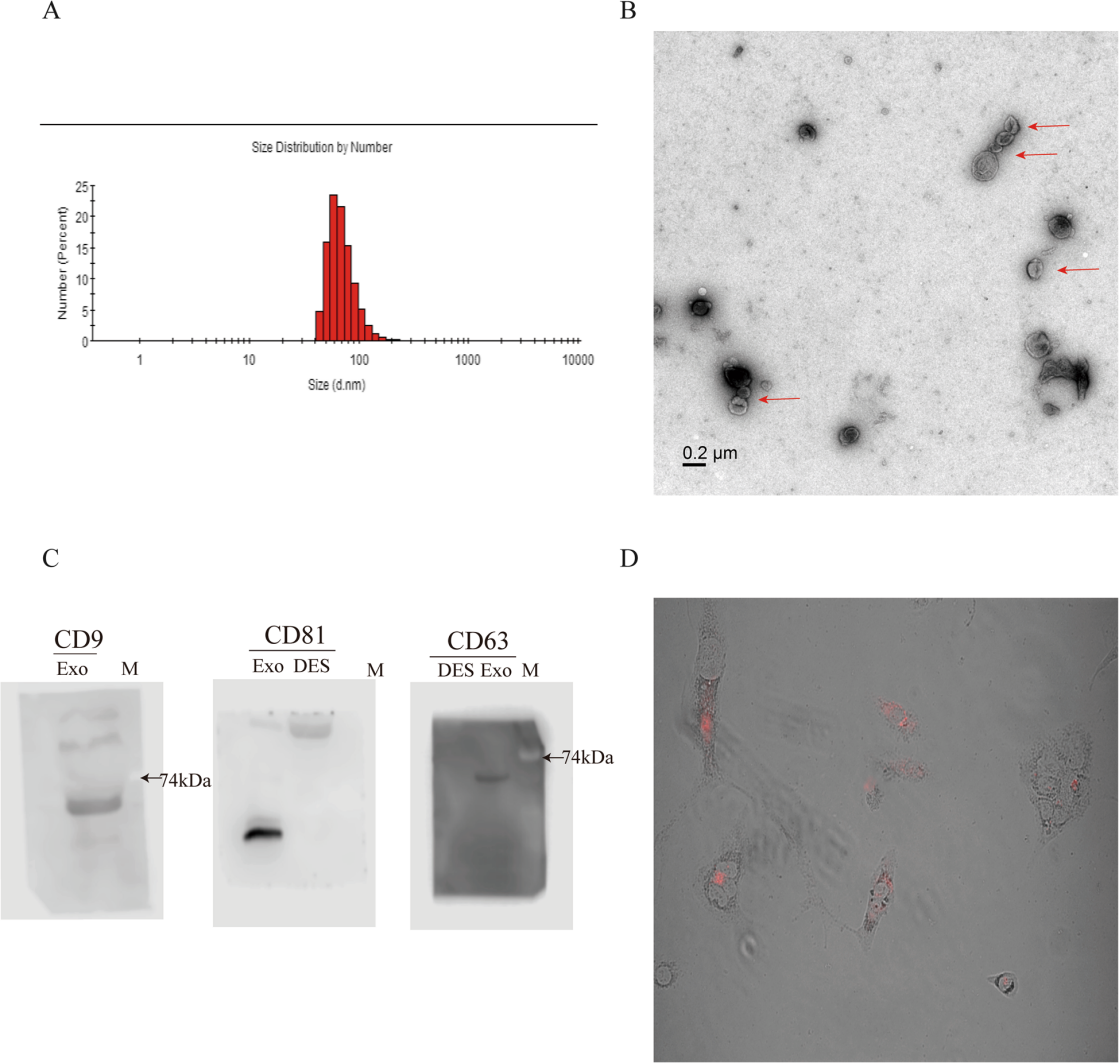
Characterization of exosomes from HCC827IR cell-derived exosomes**. a** The size distribution analysis of purified exosomes by DLS. **b** The transmission electron microscopic image of the HCC827IR cell-derived exosomes. Scale bar, 20 nm. **c** Exosome-specific markers CD9, CD81 and CD63 were detected using western blot analysis. The exosome-depleted supernatant (EDS) used as the control group. **d** The internalization of HCC827IR cell-derived exosomes in HCC827 cells. The derived HCC827IR cells

Since tumor-derived exosomes play a significant role in cancer progression [16], and combined with the above result, it was determined whether the exosomes from HCC827IR cells could affect the biological function of icotinib-sensitive lung cancer cells. The exosomes were isolated from the supernatants of HCC827IR cells, which were previously cultured for 48 h in exosome-free medium through ultracentrifugation, and kept the exosome-depleted supernatant (EDS) as the control group for subsequent experiments. The exosomes presented as rounded particles with a size of approximately 60–150 nm (Fig. 3a), and a double layer membrane, as determined by TEM (Fig. 3b). The isolated exosomes were confirmed using western blot analysis with exosome specific-markers CD9, CD63 and CD81 (Fig. 3c). The DiR-labeled exosomes were incubated with HCC827 cells for 24 h, and the localization of these exosomes was assessed by fluorescence microscopy. Then, the DiR-labeled exosomes were internalized in the cytoplasm of HCC827 cells (Fig. 3d). The result suggested that exosomes derived from HCC827IR cells were taken up by HCC827 cells.

### HCC827IR cell-derived exosomes induce the migration and invasion of HCC827 cells

**Fig. 4:**
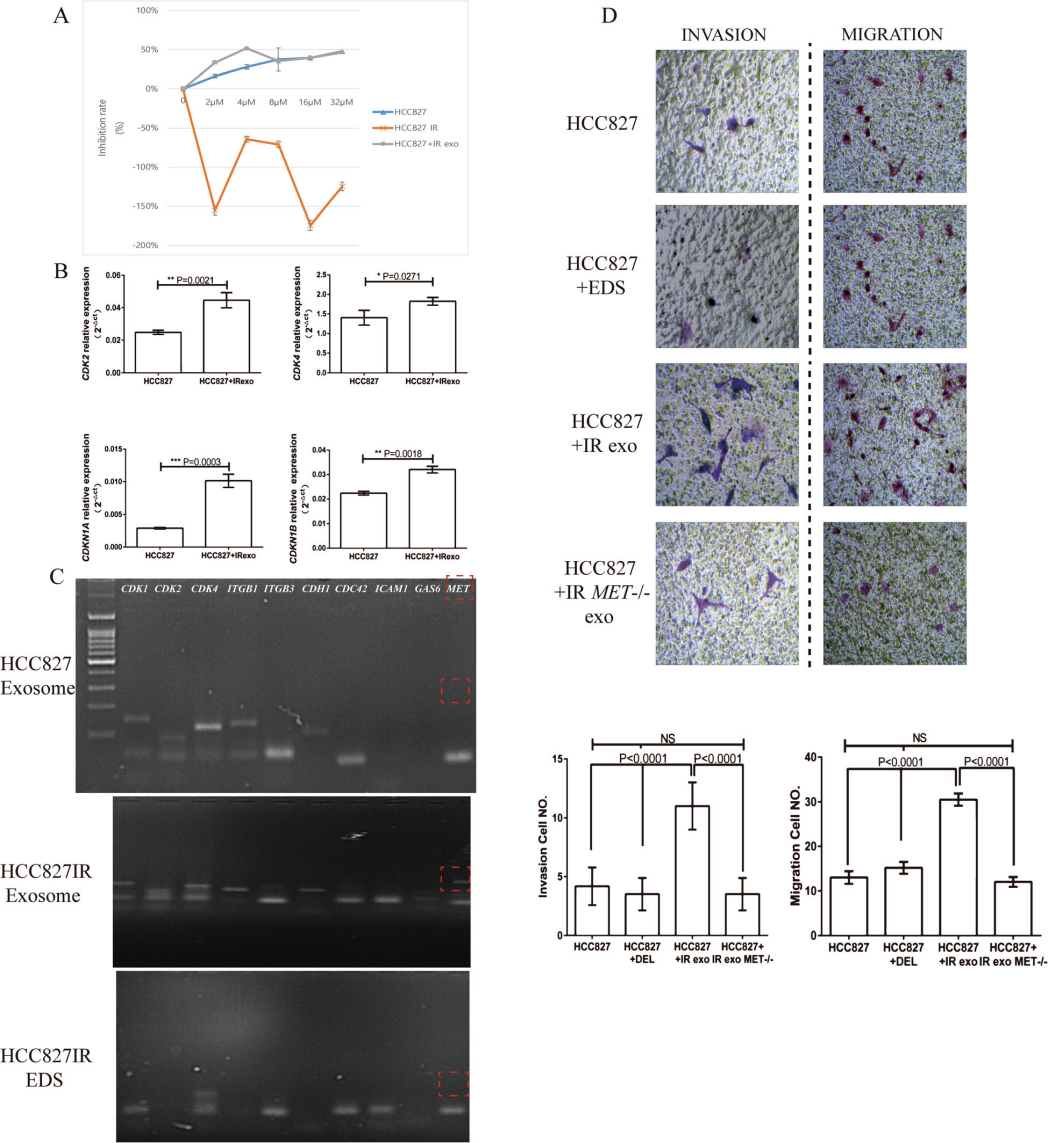
The role of HCC827IR-derived exosomes on HCC827 cells and *MET* was specifically expressed in IR exosomes. **a** MTT assay of HCC827 cells with icotinib treatment at different concentrations for 48 h after treatment with HCC827IR cells and icotinib-resistant exosomes. **b** The expression of the cell cycle and cell cycle arrest-related mRNA (*CDk2, CDk4, CDKN1a and CDKN1B*) in HCC827 and HCC827 co-cultured with icotinib-resistant exosomes was assessed by qRT-PCR. **c** The Transwell assay was performed to determine the invasion and migration ability of HCC827 cells after treatment with EDS, icotinib-resistant exosomes, and icotinib-resistant *MET*−/− exo. The migration and invasion cell numbers were analyzed by *t*-test. **d** The *MET* expression in HCC827IR exosomes was detected by PCR analysis and gel electrophoresis

In order to determine whether HCC827IR cell-derived exosomes could regulate the biological function of lung cancer cells, HCC827 cells were co-cultured with exosomes from IR cells and the EDS for 48 h, respectively. The EDS was used as a control group. The MTT assay revealed no significant differences between the treated IR exosomes and EDS on HCC827 cell proliferation (with IC50 9.2 uM, Fig. 4a). The western blot assay used to detect the expression of p-EGFR after HCC827IR-derived treatment, and the expression of p-EGFR was found not to be correlated to the icotinib concentration gradient. This shows that p-EGFR does not clearly inhibit after treatment with IR exosomes, when compared to the icotinib alone treated group (Fig. 1c). Hence, the changes in mRNA expression in IR exosomes treated HCC827 cells were further determined, which were correlated to the cell cycle distribution, using qRT-PCR analysis. The results revealed that the expression of *CDK2* and *CDK4* was significantly higher in cells treated with HCC827IR, when compared to the control group(*P* < 0.05). However, genes correlated to cell cycle arrest, such as *CDKN1A* and *CDKIB*, were also upregulated in cells treated with HCC827IR(*P* < 0.001), suggesting that IR-derived exosomes might not influence the cell cycle distribution of HCC827 cells (Fig. 4b). Furthermore, the Transwell assay revealed that the group treated with HCC827IR-derived exosomes exhibited a higher ability of migration and invasion, when compared to the EDS group (*P* < 0.001) (Fig. 4c). In order to determine the relative mRNAs loaded by exosomes involved in the increasing ability of invasion and migration of HCC827 cells, 10 candidate mRNAs relating to cell proliferation, migration and EMT were chosen to for the PCR. The results revealed that almost no free-RNA was expressed in the icotinib-resistant EDS, and most of the candidate mRNAs were loaded both in HCC827-drived exosomes and HCC827IR-drived exosomes. However, *MET* exhibited an existing band only in icotinib-resistant exosomes based on the gel electrophoresis, after staining with ethidium bromide (Fig. 4d), implying that exosomal *MET* probably plays a significant role in accelerating HCC827 migration and EMT.

### The effect of HCC827IR derived exosomal MET on HCC827 cell invasion and migration

**Fig. 5:**
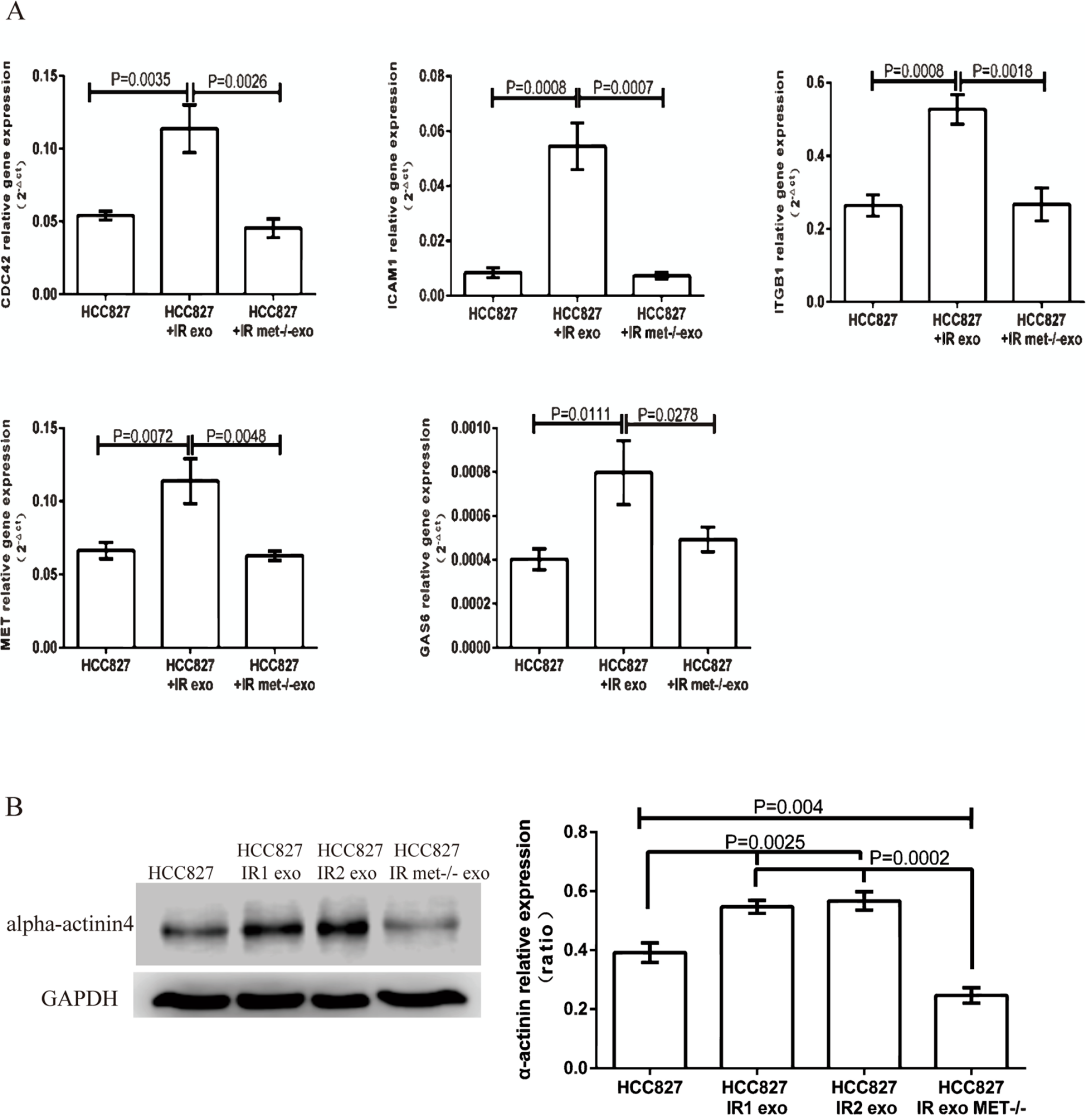
The effect of *MET* siRNA on HCC827 cells. **a** The qRT-PCR was performed to investigate the expression of *ICAM1*, *ITGB1*, *CDC42, GAS6* and *MET* in HCC827 cells treated with HCC827IR exosomes and *MET*−/− icotinib-resistant exosomes. **b** The expression of alpha-actinin 4 was analyzed by western blot, and correspondent *t*-test was performed

In order to evaluate the effects of exosomal *MET* on HCC827 cells, *MET* was knocked down in HCC827IR-derived exosomes by electroporation. The knockdown efficiency of siRNA-*MET* in exosomes was confirmed by qRT-PCR (Additional file). Then, HCC827 cells were treated with *MET*−/− icotinib-resistant exosomes, and the Transwell assay (with or without Matrigel) revealed that the migration and invasion ability of HCC827 cells incubated with *MET*−/− icotinib-resistant exosomes significantly decreased, when compared to the corresponding negative control (Fig. 4c). In addition, several mRNAs correlated to cell migration and EMT were analyzed by qRT-PCR. The results revealed a higher expression of *ICAM1*, *ITGB1*, *CDC42, GAS6* and *MET* in cells, which were co-cultured with HCC827IR exosomes and *MET*−/− icotinib-resistant exosomes (Fig. 5a). Meanwhile, the western blot analysis revealed that the expression of alpha-actinin 4 was upregulated after the treatment of icotinib-resistant exosomes, but this significantly declined after the treatment of *MET*−/− icotinib-resistant exosomes, implying that the exo-*MET* might promote HCC827 migration and invasion by upregulating alpha-actinin 4 (Fig. 5b). This result further confirms that, to some extent, the icotinib-resistant-derived exo-*MET* might attribute to the migration of lung cancer cells.

### HCC827IR cell-derived exosomes up-regulated the expression of AKT in lung epithelial cells (BEAS-2B)

**Fig. 6:**
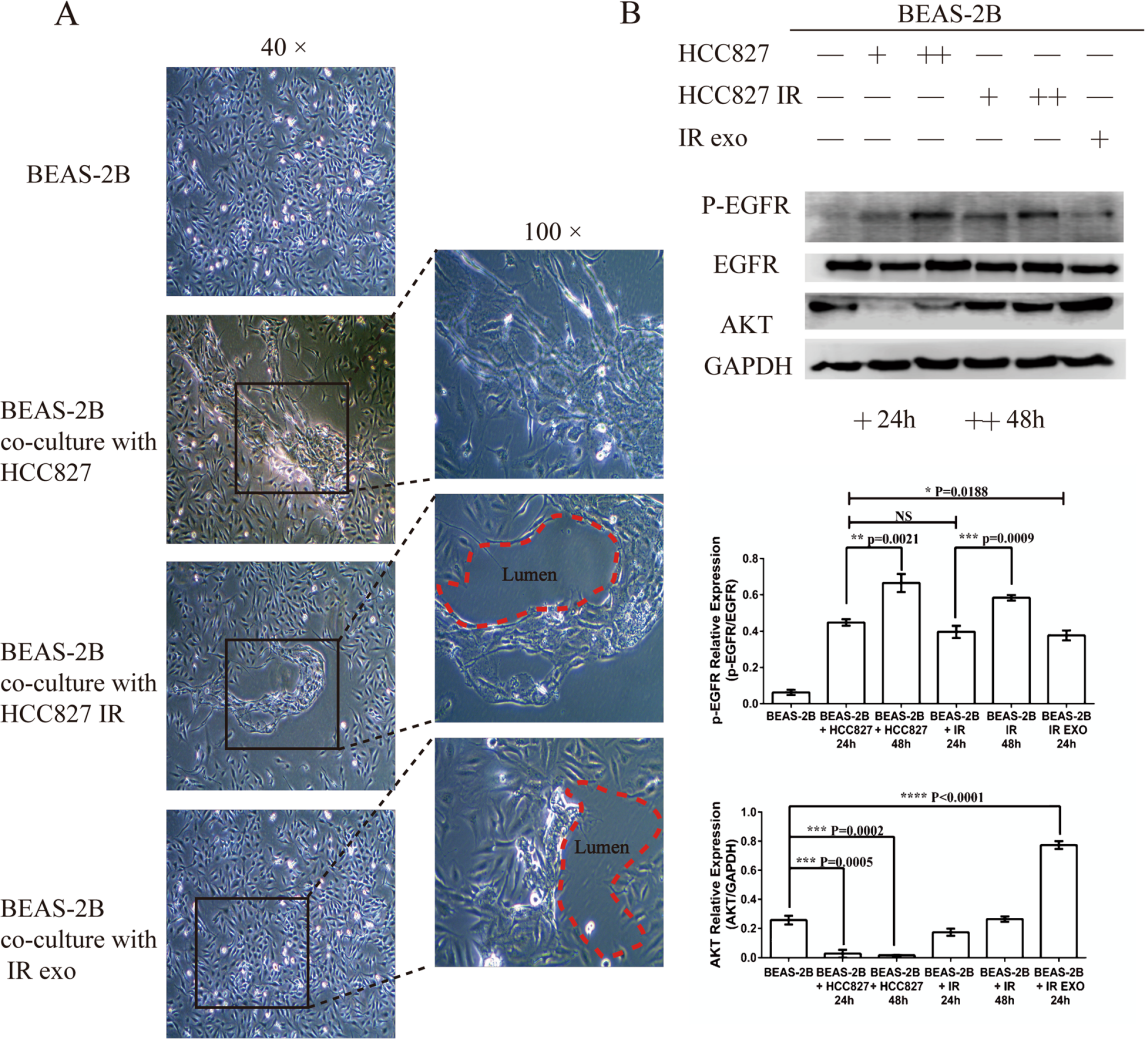
The role of HCC827IR cell-derived exosomes on lung epithelial cells (BEAS-2B). **a** The change in BEAS-2B morphology after co-culture with HCC827, HCC827IR and icotinib-resistant exosomes were observed using an optical microscope (at 40× and 100×, respectively). **b** The comparison of the expression of EGFR and P-EGFR in differently treated BESA-2B cells (including those co-cultured with HCC827, HCC827IR and icotinib-resistant exosomes)

In order to further evaluate the role of exosomes on normal lung cells, BEAS-2B lung epithelial cells were co-cultured with HCC827 cells, HCC827IR cells and HCC827-derived exosomes for 24 and 48 h, respectively, and BEAS-2B cells were cultured in normal culture medium as the control group. Initially, the changes in BEAS-2B cell morphology was observed under microscopy after culturing for 48 h (Fig. 6a). In all three groups, some of the BEAS-2B cells were observed to elongate, but marker identification of fibrosis was not performed. However, some lacuna appeared only in the intercellular space of BEAS-2B, which co-cultured with icotinib-resistant and icotinib-resistant exosomes cells (Fig. 6a). Afterwards, the protein expression was analyzed by western blot. It is noteworthy that the phosphorylation status of EGFR in BEAS-2B markedly increased in all three groups. In addition, the expression at 48 h was higher than that at 24 h. Meanwhile, in the HCC827 co-cultured group, the AKT level increased with the extension of co-culture time. However, the AKT level was significantly up-regulated in the HCC827IR and icotinib-resistant exosomes co-cultured group (b).

### The expression of exosomal MET was associated with lung cancer metastasis in NSCLC patients

**Fig. 7:**
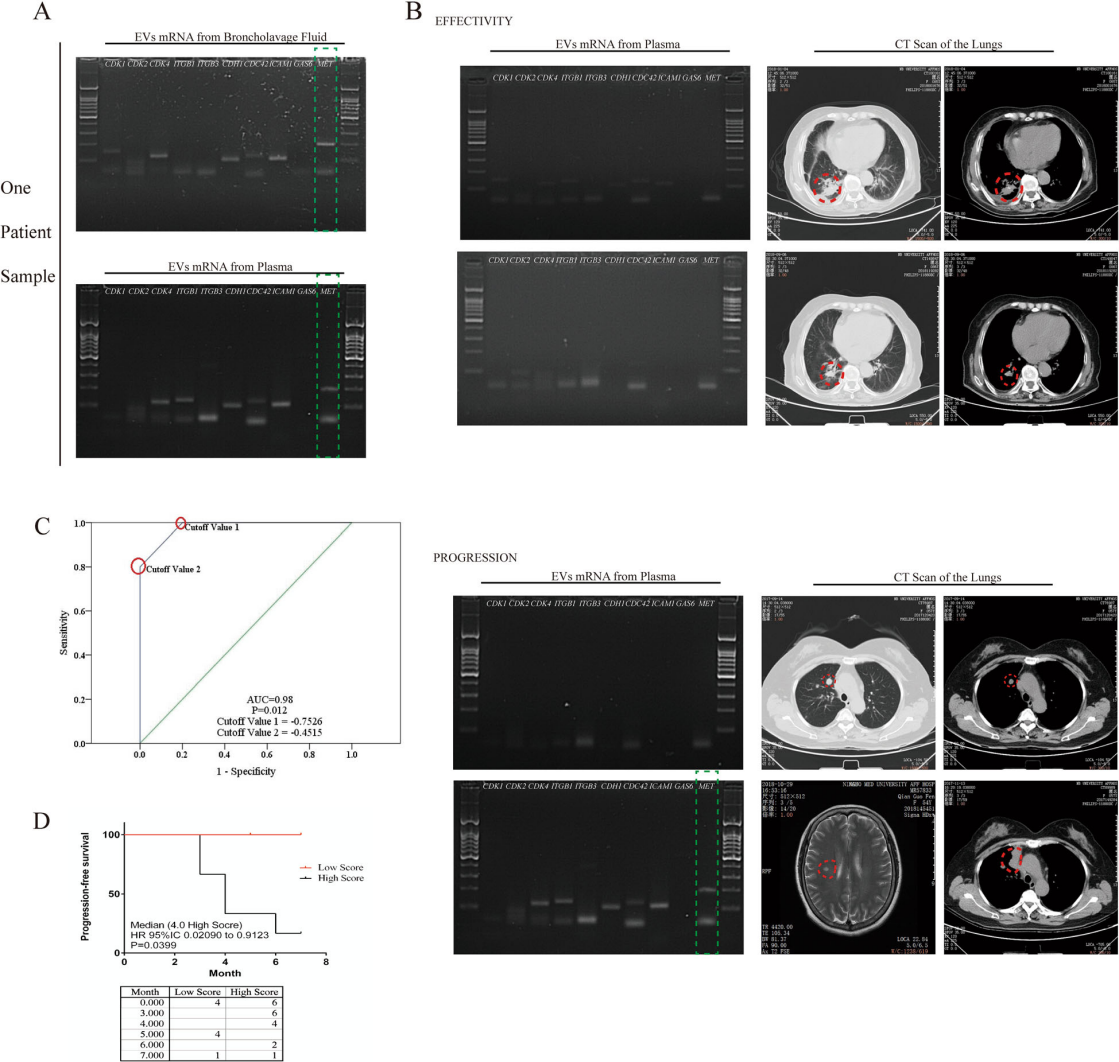
**a** Compared with the exosome mRNA obtained from BALF with that from plasma, it was found that almost all mRNAs were equally expressed in two types of body fluids, including *MET*. **b** In comparing icotinib effectivity in a patient’s plasma exo-mRNA with icotinib-resistant patients, it was found that exo-*MET* was specifically expressed in icotinib-resistant patients. The CT scan revealed the different processes of the disease after icotinib treatment. **c** The sensitivity and specificity of score of formula through the ROC curve: AUC = 0.98, *P* = 0,012, Cut-off value 1 = − 0.7526, and cut-off value 2 = − 0.45115. **d** The formula log_10_2^Score 2nd-Score 1st^ was used to calculate the relationship between the 10 candidates’ exo-mRNAs and the disease progression by PFS. (median of high score was 4, *P* = 0.0399)

The clinical characteristics of NSCLC patients (*n* = 10) are presented in Table 1. These 10 patients were similar in pathological diagnosis, but different in neoplasm stage, and treated with icotinib (except for two patients who were treated with gefitinib). The average follow-up period was approximately 3–6 months. In order to investigate the expression of the tumor cell-cycle and migration-related genes in plasma exosomes from patients with NSCLC, 10 different genes were detected, which were mentioned above, in order to compare the expression at different time points after icotinib treatment. According to PCR and gel electrophoresis analysis, it was found that the expression of 10 candidate mRNAs was the same in plasma exosomes as it was in BALF-derived exosomes (Fig. 7a), and *MET* was found to be differentially presented, with correspondent bands in four of 10 patients after treatment with icotinib for three or more months. However, these were not exhibited in lung cancer patient plasma exosomes at the previous three months (Fig. 7b). Furthermore, combined with the CT scan results, these four patients resulted in not only no efficiency of icotinib treatment, but also presented with cancer metastasis (Fig. 7b). The expression level of 10 candidate mRNAs (Table 2) in the exosomes of plasma collected from NSCLC patients at the time of primary diagnosis and the second check during the 3–6 months follow-up period was assessed. A score of 1 point represents the expression of one mRNA. Based on this scoring criteria, the difference value of 10 mRNA expression at two different time points was calculated before and after icotinib treatment, respectively. Then, these were further calculated using the formula (log_10_2^Score 2nd-Score 1st^) to evaluate the correlation of this formula and cancer progression in patients. The sensitivity and specificity of the formula were analyzed for the assessment of lung cancer cell metastasis through the receiver operating characteristic (ROC) curve analysis. This analysis revealed that the area under the curve (AUC) was 0.98, two cutoff value were − 0.7526 and − 0.4515, respectively, meaning the sensitivity and specificity of formula were significantly higher(*p* = 0.012), suggesting that the score of this formula could be used as a potential indicator for icotinib resistance in NSCLC treatment (c). Meanwhile, we used progression-free survival curve to calculate this result of formula, suggesting that the score was positively correlated with cancer progression (Median was 4, *P*-value = 0.0399, Fig. 7d).

**Table 1:** General information

SAMPLE NUMBER	GENDER	AGE	PATHOLOGICAL DIAGNOSIS	1st RECORD			TREATMENT	2nd RECORD			FOLLOW-UP PERIOD (Months)
				NEOPLASM STAGING	CEA(ug/L)	*EGFR* MUTATION		NEOPLASM STAGING	CEA(ug/L)	*EGFR* MUTATION	
5	F	85	Adenocarcinoma	IVa	102.59	*19 del*	Icotinib		18.04	*undetected*	7
14	F	57	Adenocarcinoma	IVa	6.46	*19 del*	Icotinib	IVc	3.07	*T790 m*	4
52	F	81	Adenocarcinoma	IVa	89.24	*19 del*	Icotinib	IVc	77.5	*undetected*	6
28	M	67	Adenocarcinoma	IIIa	42.63	*19 del*	Icotinib		14.99	*undetected*	7
22	F	53	Adenocarcinoma	Iva	355.92	*19 del*	Icotinib	IVc	564.25	*19 del*	5
24	M	74	Adenocarcinoma	IVa	126.78	*19 del*	Icotinib		4.85	*undetected*	7
62	M	68	Adenocarcinoma		41.25	*19 del TP53*	Gefitnib		6.42	*undetected*	5
31	F	66	Adenocarcinoma	IVa	46.73	21L858R	Icotinib	IVc	76.11	undetected	3
39	F	79	Adenocarcinoma	IVa	1025	*19 del*	Gefitnib		88.1	*undetected*	5
4	M	86	Adenocarcinoma	IVa	10.31	21L858R	Icotinib		12.33	*undetected*	4

**Table 2:** The formula

Patient number	1st Test				2nd Test							
	Follow- up period (months)	Cell cycle-related genes in EVs (NO.)	Migration-related genes in EVs (NO.)	Reprogramming-related genes in EVs (NO.)	Total Score	Cell cycle-related genes in EVs (NO.)	Migration-related genes in EVs (No.)	Reprogramming related genes in EVs (NO.)	Total Score	2^Score 2nd-Score 1st^	log_10_2^Score 2nd-Score 1st^	Tumor Progression
5	7	2	3	0	5	1	1	0	2	0.125	−0.903089987	0
14	4	0	0	0	0	1	4	1	6	64	1.806179974	1
52	6	0	1	1	2	1	3	1	5	8	0.903089987	1
28	7	1	2	1	4	0	2	0	2	0.25	−0.602059991	0
22	5	2	2	1	5	0	2	1	2	0.125	−0.903089987	1
24	7	0	2	0	2	0	1	0	1	0.5	−0.301029996	0
62	5	2	1	1	4	0	0	0	0	0.0625	−1.204119983	0
31	3	1	1	1	3	1	4	1	6	8	0.903089987	1
39	5	2	2	1	5	0	2	0	3	0.25	−0.602059991	0
4	4	1	4	0	5	1	1	0	2	0.125	−0.903089987	0

## Discussion

EGFR-targeted tyrosine kinase inhibitors (TKIs) have been widely used as a major treatment for NSCLC patients with EGFR-mutation. However, resistance to TKIs is becoming increasingly frequent, limiting its clinical efficacy. Previous studies have reported the possible causes of drug resistance, such as the tumor microenvironment and cancer cell interaction [17–19]. Exosomes, as a delivery of various biological molecules (proteins, mRNAs, miRNAs and others), have been recognized to mediate cellular communication and tumor microenvironment regulation. Accumulating evidence has revealed that tumor-derived exosomes can promote tumor progression, metastasis and drug resistance in cancer cells by transmitting exosomal contents [20–23]. In addition, previous studies have reported the relationship between exosomes and acquired drug resistance, in addition to the investigation on the underlying mechanism of drug resistance in lung cancer cells [13, 24, 25]. However, studies on the molecular and cellular mechanism of exosome-mediated metastasis of lung cancer cells have yet to be further elucidated. The present study investigated the possible effect of exosomes from icotinib-resistant lung cancer cells on icotinib sensitive lung cancer cell biological function, and it was found for the first time that eoxosmes containing *MET* may play an important role in lung cancer cell metastasis. *MET* expression was observed in HCC827 cells, which incubated with exosomes from HCC827IR. In the meantime, the ability of invasion and migration of HCC827 cells significantly improved.

*MET* is a protein coding gene that encodes a member of the receptor tyrosine kinase family of proteins and the product of the proto-oncogene *MET*. Gene amplification and the resulting over expression have been reported in several cases of patients with esophageal cancer, gastric cancer and NSCLC [26–28]. It was found earlier in some studies that *MET* amplification has a central role in acquired resistance to EGFR tyrosine kinase inhibitor therapy in EGFR-mutant NSCLC [29, 30]. In the present study, by comparing the expression of 10 candidate genes in exosomes obtained from icotinib-resistant lung cancer cells, *MET* was found to be the significantly expressed one. However, when icotinib sensitive parental cells were co-cultured with HCC827IR cells and icotinib-resistant cell-derived exosomes, it was observed that instead of inducing icotinib resistance in parental cells, *MET* expression was correlated to the increasing ability of migration and invasion of lung cancer cells. The role of the *MET* oncogene in mediating cellular transformation and tumor cell motility, invasion and metastasis has been reported in various studies [31–33]. For example, *Hector at el.* reported that the transmission of the MET onco-protein from tumor-derived exosomes to bone marrow progenitor cells promote the metastatic process [34]. Combining the present results with previous studies, it could be hypothesized that exosomes that carried *MET* in lung cancer cells with icotinib-acquired resistance may be associated with lung cancer invasiveness and metastasis.

In order to further verify this hypothesis, the exosomes were isolated from the plasma and BALF of NSCLC patients before and after icotinib treatment, and the differences in exosomes were analyzed. In agreement with the previous results of the investigators, *MET* was detected in exosomes from patients diagnosed with metastasis, according to the CT scan result at the time of the second or third follow-up examination. In addition, the same expression of the 10 candidate mRNAs was observed in plasma- and BALF-derived exosomes (Fig. 7a). BALF has been considered to be a sample that best represents the condition of pulmonary diseases, except for biopsy. However, BALF can only be obtained through the invasive examinational pathway. Hence, patients may suffer from existing pain after the examination. Therefore, it was considered that observing the same expression of the above mRNAs both in plasma and BALF indicate that plasma may be a reliable substitute for BALF to investigate the potential biomarker in a less invasive approach. It is generally known that the development of diseases is processed through multiple reasons. Hence, the single factor candidate biomarker cannot be applied as a high-quality diagnostic tool. In the present study, a formula was established by calculating the expression level of 10 exo-mRNAs in the plasma of NSCLC patients, in order to assess the process of NSCLC patients treated with icotinib. The investigators consider that this formula could be a potential prediction tool based on a small sample size. Therefore, the efficiency of this formula would be verified as a reliable diagnostic method in clinic by collecting more samples in future studies.

Admittedly, the underlying molecular mechanism of exosomal *MET*-mediated cancer invasiveness and metastasis has been widely investigated, and other *MET* involved pathways have been studied in some cancers, such as gastric cancer [6], pancreatic cancer [35], and breast cancer [36]. Although electroporation was utilized to transplant *MET* siRNA into the icotinib-resistant exosome, which was observed for the first time to block the cell-cell communication by downregulating the mRNA in exosomes. Nevertheless, it remains unclear whether the expression of exsomal *MET* is responsible for the metastasis in icotinib-treated NSCLC. Thus, the further understanding on the role of icotinib-resistant lung cancer cell-derived exosomes is needed to clarify the possible correlation between the relative pathway and exosomal *MET* expression. The present study has several limitations. First, in spite of the achievement of the high sensitivity and specificity of the score getting from the formula indicating its role as a potential marker for metastasis, the amounts of clinical patients and samples were fewer than what was originally planned. Thus, it is necessary to expand the sample size to make the result more reliable. Second, the protein expression level of *MET* was not detected, which needs to be further investigated. Third, the knockdown effect of *MET* was detected only in exosomes, and should be performed in icotinib-resistant cell line to be further confirmed.

## Conclusion

Drug resistance after the treatment of NSCLC based on EGFR-TKI target drugs remains as a challenging problem. In the present study, it was found that icotinib-resistant EGFR 19del cell HCC827 could derive exosomal MET(exo-*MET*) to induce parental cell migration and invasion. This suggests that intercellular communication leading to tumor escape may be an ideal approach for targeted drug resistance.

## Supplementary information

**Additional file 1: Table S1.** qRT-PCR primers sequence **Table S2.** MET siRNA sequence **Figure S1.** After MET siRNA was transfected into IR exosomes via electroporation, the expression of MET mRNA was detected by electrophoresis and qRT-PCR. ELE: electroporation. IR exo: HCC827 Icotinib resistance exosome.

## Abbreviations

EDS: Exosome-depleted supernatant
EMT: Epithelial-mesenchymal transition
IC50: Half maximal inhibitory concentration
IR: Icotinib resistance
NSCLC: Non-small cell lung cancer
