# Supplementary material for: Icotinib-resistant HCC827 cells produce exosomes with mRNA MET oncogenes and mediate the migration and invasion of NSCLC
Source: Respir Res. 2019 Oct 12;20:217. doi: 10.1186/s12931-019-1202-z (PMC6790059; doi:10.1186/s12931-019-1202-z)
Supplement: Supplementary file 1 — Additional file 1: Table S1. qRT-PCR primers sequence Table S2. MET siRNA sequence Figure S1. After MET siRNA was transfected into IR exosomes via electroporation, the expression of MET mRNA was detected by electrophoresis and qRT-PCR. ELE: electroporation. IR exo: HCC827 Icotinib resistance exosome. [file 12931_2019_1202_MOESM1_ESM.docx]

| mRNA name | Sequence 5’ to 3’ |
| --- | --- |
| CDC42 F | CCATCGGAATATGTACCGACTG |
| CDC42 R | CTCAGCGGTCGTAATCTGTCA |
| ICAM1 F | ATGCCCAGACATCTGTGTCC |
| ICAM1 R | GGGGTCTCTATGCCCAACAA |
| ITGB1 F | CAAGAGAGCTGAAGACTATCCCA |
| ITGB1 R | TGAAGTCCGAAGTAATCCTCCT |
| ITGB3 F | AGTAACCTGCGGATTGGCTTC |
| ITGB3 R | GTCACCTGGTCAGTTAGCGT |
| CDK4 F | ATGGCTACCTCTCGATATGAGC |
| CDK4 R | CATTGGGGACTCTCACACTCT |
| CDK2 F | CCAGGAGTTACTTCTATGCCTGA |
| CDK2 R | TTCATCCAGGGGAGGTACAAC |
| CDK1 F | AAACTACAGGTCAAGTGGTAGCC |
| CDK1 R | TCCTGCATAAGCACATCCTGA |
| CDH1 F | ATTTTTCCCTCGACACCCGAT |
| CDH1 R | TCCCAGGCGTAGACCAAGA |
| CDKN1B F | TAATTGGGGCTCCGGCTAACT |
| CDKN1B R | TGCAGGTCGCTTCCTTATTCC |
| CDKN1A F | TGTCCGTCAGAACCCATGC |
| CDKN1A R | AAAGTCGAAGTTCCATCGCTC |
| met F | AGCAATGGGGAGTGTAAAGAGG |
| met R | CCCAGTCTTGTACTCAGCAAC |
| GAS6 F | CTCGTGCAGCCTATAAACCCT |
| GAS6 R | TCCTCGTGTTCACTTTCACCG |

1、QPCR primers

2、Met siRNA sequence 5’ to 3’

Sense: GGUGUUGUCUCAAUAUCAATT

Anti-sense: UUGAUAUUGAGACAACACCTT

3、met siRNA electroporation


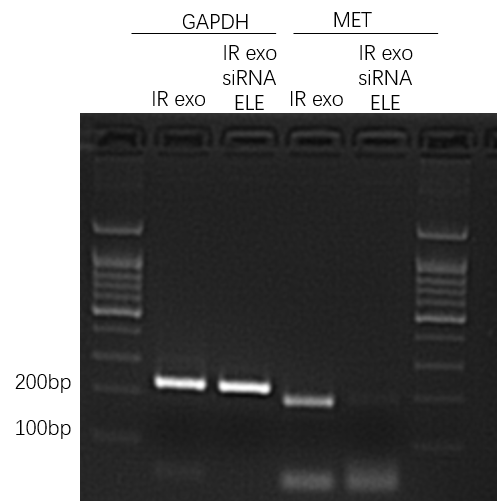




ELE：electroporation. IR exo: HCC827 Icotinib resistance exosome
